# Elbow valgus stability of the transverse bundle of the ulnar collateral ligament

**DOI:** 10.1186/s12891-021-04760-1

**Published:** 2021-10-12

**Authors:** Mutsuaki Edama, Kanta Matsuzawa, Hirotake Yokota, Ryo Hirabayashi, Chie Sekine, Sae Maruyama, Noboru Sato

**Affiliations:** 1 Institute for Human Movement and Medical Sciences, Niigata University of Health and Welfare, Niigata, Shimami-cho 1398, Kita-ku, Niigata City, 950-3198 Japan; 2 Division of Gross Anatomy and Morphogenesis, Niigata University Graduate School of Medical and Dental Sciences, Niigata, Japan

**Keywords:** Thiel-embalmed cadavers, Gross anatomy, Elbow joint, Ultrasonography

## Abstract

**Background:**

The purpose of this study was to clarify elbow valgus stability of the transverse bundle (TB). We hypothesized that the transverse bundle is involved in elbow valgus stability.

**Methods:**

Twelve elbows of six Japanese Thiel-embalmed cadavers were evaluated. The skin, subcutaneous tissue and origin of forearm flexors were removed from about 5 cm proximal to the elbow to about 5 cm distal to the elbow, and the ulnar collateral ligament was dissected (intact state). The cut state was defined as the state when the TB was cut in the middle. The joint space of the humeroulnar joint (JS) was measured in the intact state and then in the cut state. With the elbow flexed to 30°, elbow valgus stress was gradually increased to 30, 60 N using the Telos Stress Device, and the JS was measured by ultrasonography under each load condition. Paired t-testing was performed to compare the JS between the intact and cut states under each load.

**Results:**

No significant difference in JS was identified between the intact and cut state at start limb position. The JS was significantly higher in the cut state than in the intact state at both 30 N and 60 N.

**Conclusion:**

The findings from this study suggested that the TB may be involved in elbow valgus stability.

## Introduction

There is currently insufficient evidence in the literature to establish statistically significant differences in the effects of conservative versus surgical treatment on the functional outcomes of patients with the ulnar collateral ligament (UCL) lesions [1]. Therefore, it is important to clarify the functional role of UCL.

The UCL consists of an anterior bundle (AB), posterior bundle, and transverse bundle (TB) [2–4]. The AB has been reported as a primary stabilizer for elbow valgus stress, with the posterior bundle as a secondary stabilizer [5–9]. Many anatomical studies [3, 4, 7, 10–24] and biomechanical studies [25–30] have been reported on AB and PB. But there are few anatomical studies and biomechanical studies on TB [2, 20, 31, 32]. The TB has been thought to potentially assist function of the AB, as this bundle is continuous with the AB [2, 20, 31, 32]. On the other hand, the TB has not been thought to be involved in elbow valgus stability [10, 33–35], because the bundle does not cross the humeroulnar joint [31]. Ciccotti et al. [36] performed a study that divided fresh cadavers into two groups, measuring the joint space of the humeroulnar joint (JS) using ultrasonography before and after cutting soft tissues [36]. As a result, one group showed significantly increased JS after cutting the TB, but no significant difference was seen in the other group. These suggested that the contribution of the TB to elbow valgus stability has not been sufficiently investigated. In addition, that study examining elbow valgus stability had not specifically focused on the TB, and specimen conditions may differ due to the order in which soft tissues were cut in different groups [36]. Therefore, we believe that focusing on the TB is important, and evaluation and comparison under the same conditions is needed to clarify elbow valgus stability of the TB.

The purpose of this study was to clarify elbow valgus stability of the TB. We hypothesized that the TB is involved in elbow valgus stability.

## Methods

### Cadavers

Twelve elbows from six Japanese Thiel-embalmed cadavers (mean age at death, 87.8 ± 5.8 years; 4 males, 2 females) donated to the university anatomy program were evaluated. Thiel embalming has been reported as a fixation method that can maintain flexibility in a life-like condition [37, 38], facilitating reliable ultrasound imaging [39, 40] and life-like mechanical properties [37, 39, 40]. In addition, Thiel-embalmed cadavers reportedly show JS changes similar to those seen in living human volunteers when elbow valgus stress is applied [41]. Specimens with deformation of the elbow joint or a history or elbow surgery were excluded. This study was performed in accordance with the Declaration of Helsinki after all protocols were approved by the ethics committee of our institution.

### Measurement conditions

**Fig. 1:**
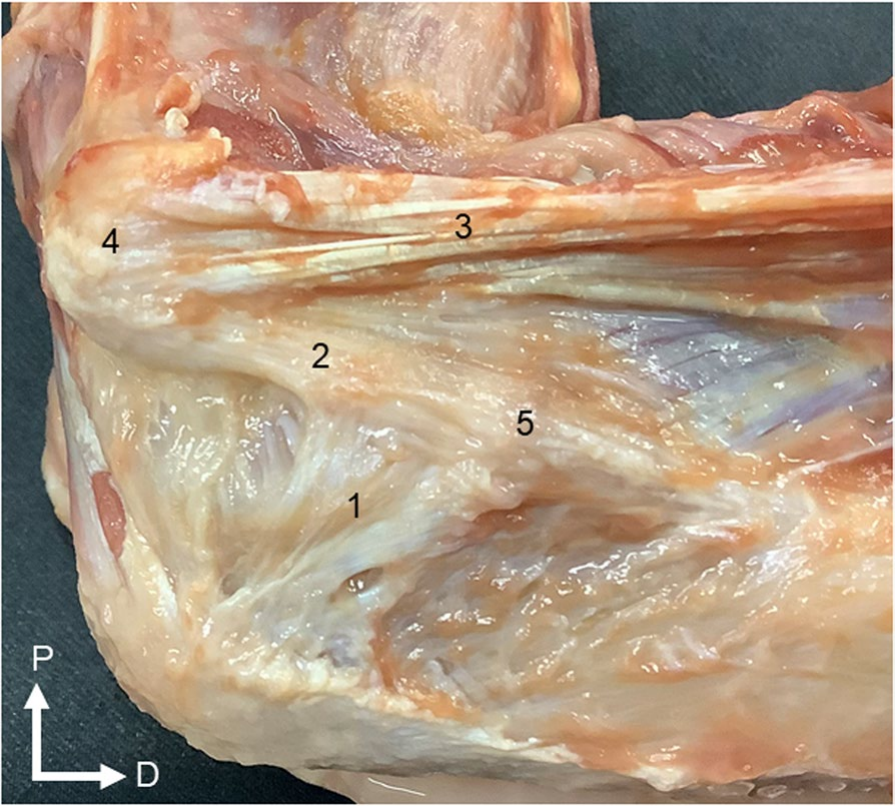
Dissection of the transverse bundle of the ulnar collateral ligament (left elbow, medial view). 1: Transverse bundle of the ulnar collateral ligament; 2: anterior bundle of the ulnar collateral ligament; 3: anterior common tendon; 4: medial epicondyle of the humerus; 5: sublime tubercle of the ulna. P: proximal; D: distal

For dissection, the skin and subcutaneous tissues were removed from about 5 cm proximal to the elbow to about 5 cm distal to the elbow. The origins of the pronator teres, flexor carpi radialis, flexor digitorum superficialis and flexor carpi ulnaris muscles were removed, and the UCL was dissected [32]. The ulnar nerve was cut, the anterior common tendon was not removed, but the posterior common tendon was removed (Fig. 1). The intact state was defined as the state in which the UCL had been dissected, while the cut state was defined as the state when the TB had been cut in the middle. The JS was measured in the intact state, and then in the cut state, with the interval between measurements set at 15 min.

### Measurement of the joint space in the humeroulnar joint

**Fig. 2:**
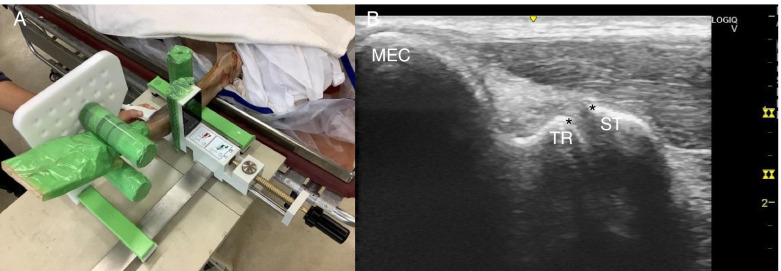
Measurement position for ultrasonic imaging. **A** Measurement position of the Thiel cadaver. The Thiel cadaver is placed in the supine position with the shoulder in abduction and external rotation of 90°, and the elbow at 30° of flexion and forearm supination in the Telos stress device. **B** Long-axis image of the humeroulnar joint. MEC: medial epicondyle of the humerus; TR: trochlea of the humerus; ST: sublime tubercle of the ulna

**Fig. 3:**
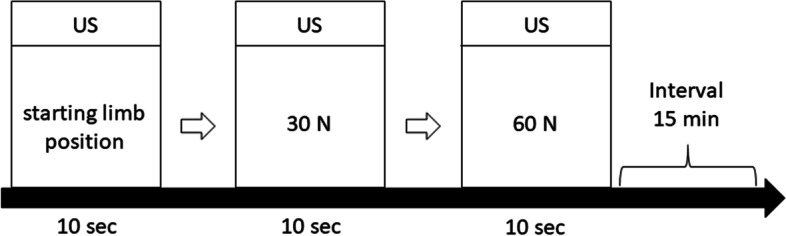
Measurement protocol of the joint space of the humeroulnaris joint. First, the Telos device is set to starting limb position, and three images of the medial part of the elbow are taken. The load is then gradually increased at 10 N/sec, and when 30 N and 60 N are reached, three ultrasonic images are taken within 10 s at each point. US: Take three ultrasound images. White arrows: the load is gradually increased at 10 N/sec

This study referred to a previous study [41] that measured the JS. Thiel-embalmed cadavers were placed in the supine position, with the shoulder abducted and in 90° of external rotation, with the elbow flexed to 30° and the forearm supinated in a Telos Stress Device (Telos) (Aimedic MMT, Tokyo, Japan). JS measurement was performed using ultrasonography (LOGIQ eV2; GE Healthcare, Qujing, China) with a 12-MHz linear probe. The probe was placed on the medial side of the elbow joint, and AB was visualized using the humeral trochlea and sublime tubercle of the ulna as landmarks (Fig. 2A). Using the built-in calipers of the ultrasonography device, the JS was measured as the distance between the distal-medial corner of the humeral trochlea and the proximal edge of the sublime tubercle of the ulna (Fig. 2B). Sufficient the intraclass correlation coefficient (ICC) has been confirmed for the US measurement [41]. US measurement was performed by 1 operator (M.E.) and the Telos was performed by another operator (K.M.). This study also referred to a previous study [41] for the measurement protocol. The Telos was first set to starting limb position, and three images of the medial elbow were taken. The load was then gradually increased at + 10 N/s, and three ultrasonic images were taken within 10 s when 30 N was reached, and again when 60 N was reached. Mean values of the JS from the three images were used (Fig. 3).

### Statistical analysis

To examine the elbow valgus stability of the TB, paired t-testing was performed for the JS of the intact and cut states under each load. Differences were considered significant at the 5% level.

## Results

No significant differences in JS were seen between intact and cut states at starting limb position. The JS was significantly higher in the cut state than in the intact state at both 30 N (*P* = 0.044) and 60 N (*P* = 0.004) (Table 1).

**Table 1:** Joint space of the humeroulnar joint at each load volume

	Intact state	Cut state	*P* value
Starting limb position	4.03 ± 0.69	3.97 ± 0.63	0.238
30 N	4.31 ± 0.54	4.41 ± 0.65	0.044
60 N	4.62 ± 0.59	4.77 ± 0.62	0.004

Values are expressed in millimeters as means ± standard deviations

Intact state: The ulnar collateral ligament was dissected

Cut state: The transverse bundle of the ulnar collateral ligament was cut in the middle

## Discussion

This study focused on the TB and examined elbow valgus stability under different conditions of the TB using Thiel-embalmed cadavers. To the best of our knowledge, no previous studies have focused on the TB and examined elbow valgus stability of the TB.

In this study, no significant difference in JS size between intact and cut states was seen at starting limb position, but the JS was significantly larger in the cut state than in the intact state at both 30 N and 60 N. This study therefore suggested that the TB may be involved in elbow valgus stability. A previous study reported that osseous, capsule, and the UCL are involved in elbow valgus stability [8]. Among these, the AB was reported to be the most involved in elbow valgus stability [7]. Previous studies have reported that the TB is continuous with the AB in all specimens [2, 20, 32, 35]. The TB may thus be secondarily involved in elbow valgus stability. This study suggests that it may provide useful basic data for the diagnosis and reconstruction of UCL injury.

This study has several limitations. First, this study is that the TB could not be classified based on morphology because of the small number of specimens. In the previous studies, the TB was classified as follows TB does not continue the entire length of the AB or TB continues the entire length of the AB [2, 32]. Further study of a larger number of specimens is thus needed, to examine whether the function of the TB differs depending on morphology. Second, it has been reported that the mechanical properties of Thiel cadaver are not similar to life-like, depending on the measurement part [42]. But, in a previous study, it was revealed that the changes in JS associated with increased elbow valgus stress are similar to those in human volunteers [41]. Third, this study is that the dominant hand of the donors was unknown, and whether donors had been involved in overhead sports during their lifetime was also unknown. A previous study reported that the difference in the JS between valgus stress and no valgus stress was significantly higher in the dominant arm than in the non-dominant arm [43]. A previous study also reported that the JS of professional pitchers was significantly greater in the dominant arm than in the non-dominant arm [44]. In the present study, the numbers of left and right samples were balanced as much as possible to minimize these effects. Therefore, the effects of the dominant arm and involvement in overhead sports were thought to have been minimized.

## Conclusions

The findings of this study suggest that the TB may be involved in elbow valgus stability.

## Data Availability

The datasets generated and/or analysed during the current study are not publicly available due to limitations of ethical approval involving the patient data and anonymity but are available from the corresponding author on reasonable request.

## Abbreviations

AB: anterior bundle
JS: humeroulnar joint
TB: transverse bundle
Telos: Telos Stress Device
UCL: ulnar collateral ligament
